# Endodontic Retreatment of a Mandibular Second Molar With a C-shaped Root Canal Configuration: A Case Report

**DOI:** 10.7759/cureus.52812

**Published:** 2024-01-23

**Authors:** He Liu, Ya Shen

**Affiliations:** 1 Division of Endodontics, Department of Oral Biological and Medical Sciences, Faculty of Dentistry, University of British Columbia, Vancouver, CAN

**Keywords:** dental operating microscope, bioceramic sealer, c-shaped root canal configuration, mandibular second molar, root canal retreatment

## Abstract

Root canal retreatment in mandibular second molars with C-shaped root canal configurations presents notable challenges. This article presents a case of successful root canal retreatment in a mandibular second molar exhibiting this complex configuration. Achieving a successful endodontic outcome in such cases necessitates a comprehensive understanding of the unique root canal anatomy. Moreover, the employment of advanced instruments and techniques is crucial to effectively address the intricacies of the C-shaped root canal system.

## Introduction

Bacterial biofilm, a sophisticated community of microorganisms attached to surfaces and encased in a self-produced extracellular matrix, is the predominant cause of pulp tissue inflammation and apical periodontitis [1-3]. Root canal treatment stands as the principal strategy to combat pulp tissue inflammation and bacterial infection within a tooth's root canal system [4]. The core objective of this treatment is the thorough removal of bacterial biofilm and infected tissue from the pulp space. By doing so, it aims to halt the progression of infection and foster the healing of both pulpal and periapical tissues [4]. The efficacy of root canal treatment largely hinges on meticulous instrumentation [5-6], irrigation [7-9], disinfection [10], and subsequently filling the root canal system [11-13]. These steps are crucial not only for eradicating the infection but also for preserving the tooth's functionality and integrity.

Despite a generally high success rate, root canal treatment does not always guarantee success. Failure rates, varying between 15 and 25%, depend on the stringency of the success criteria used, whether it is the complete resolution (strict criteria) or just a reduction in the size of a periapical lesion (loose criteria) [14]. Numerous studies in endodontics have focused on identifying risk factors that influence treatment outcomes, categorizing them into three main areas: pre-operative, intra-operative, and postoperative factors [15-19]. Pre-operative factors include patient demographics, systemic health, tooth characteristics and root canal anatomy, diagnosis, and the presence of periapical lesions [15]. Intra-operative factors encompass the techniques used in chemomechanical preparation, the quality of root canal filling, and the operator's experience, skills, and protocol [15]. Postoperative factors involve the quality of the restoration done after the procedure [15]. Notably, the homogeneity and apical extension of the root canal filling play a critical role in the healing or persistence of periapical lesions following endodontic treatment [19].

When root canal treatment fails, nonsurgical retreatment is often the first option [20, 21]. A recent systematic review and meta-analysis revealed that the success rate of non-surgical root canal retreatment is around 71% with strict criteria and 87% with loose criteria over 1-3 years, and 77% with strict criteria over 4-5 years [14]. Factors like periapical radiolucency, larger periapical lesions, higher initial periapical index (PAI) scores, multiple-visit retreatments, and certain tooth types (such as mandibular molars) are associated with lower success rates [14].

Mandibular second molars typically have two roots and three canals but can exhibit variations such as extra roots or canals [22-24]. One specific variation is the C-shaped canal configuration [22-24]. The C-shaped root canal configuration, predominantly found in mandibular second molars, arises primarily due to the incomplete fusion of Hertwig’s epithelial root sheath on either the lingual or buccal root surface. This developmental anomaly results in a root that invariably encompasses a C-shaped canal. Additionally, the formation of a C-shaped root can also occur through a process of coalescence, where the deposition of cementum over time leads to the merging of initially separate canals into a single C-shaped configuration [22-24]. A study using cone-beam computed tomography (CBCT) identified an average prevalence of 12% for C-shaped canals in mandibular molars, with gender and geographic region influencing prevalence [24]. 

A number of studies have focused on categorizing C-shaped roots and root canals, utilizing various techniques such as transverse sectioning, visual examination, histological analysis, and X-ray imaging [22-24]. Melton et al. conducted a study on 15 extracted mandibular second molars, applying the resin cast method and histological sectioning to inspect the root canals [22]. Their research led to the development of a streamlined classification system for C-shaped root canals, comprising three distinct types. Type I features a continuous C-shaped canal without any separations. Type II is characterized by a semicolon-shaped canal, and Type III represents cases where the tooth possesses two or more distinct canals. This classification system, or variations inspired by it, has been widely adopted in subsequent research [22].

C-shaped root canals pose unique challenges in endodontic treatment due to their complex anatomy, often featuring a single ribbon-like space or an isthmus connecting the canals [25-28]. A study by Kim et al. focused on C-shaped root canals, predominantly in mandibular second molars, analyzing the causes of endodontic failure. They found that the most common reasons for failure included leaky canals and issues with the isthmus, as well as missed canals, overfilling, and iatrogenic problems [25].

Retreating mandibular second molars with previously failed root canal treatments demands meticulous removal of all filling materials and thorough reshaping, recleaning, redisinfecting, and refilling of the root canal system [25-28]. This article highlights a successful case of root canal retreatment in a mandibular second molar with a C-shaped canal configuration. The complexity of such cases underscores the importance of advanced endodontic techniques and a deep understanding of tooth anatomy for effective treatment.

## Case presentation

**Figure 1:**
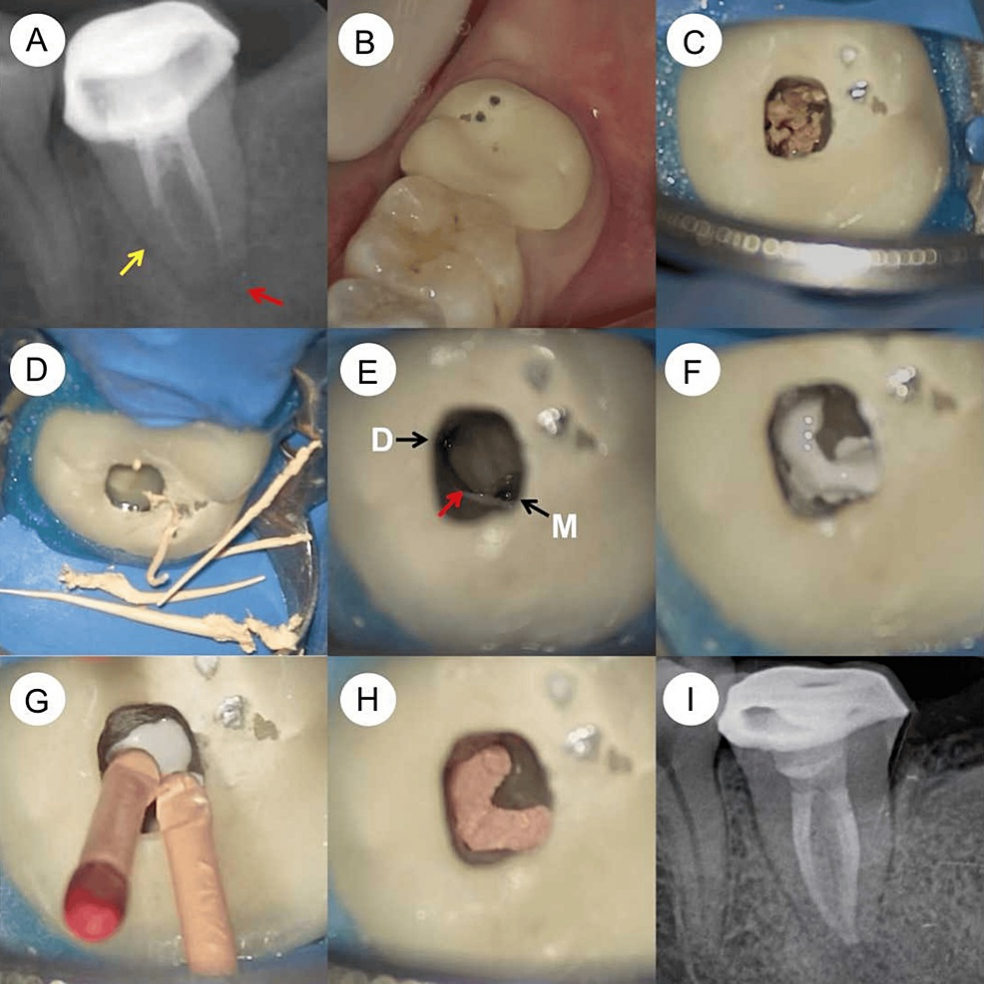
Periapical radiographs and microscopic photographs were taken during the retreatment procedures of the mandibular second molar, which has a C-shaped root canal configuration. (A) A preoperative periapical radiograph reveals substandard quality in the previous root canal treatment of tooth #37, along with a radiolucent area surrounding its apex (red arrow). The single conical root (yellow arrow) suggests a C-shaped root canal configuration in tooth #37. (B) A microscopic image displays the partial chipping of the porcelain crown on tooth #37, revealing its metal base. (C) Another microscopic image illustrates the exposed pulp chamber floor of tooth #37, with remnants of gutta-percha present in the chamber. (D) The gutta-percha residues are meticulously removed using a #20/.02 Micro-Debrider (Dentsply Maillefer, Ballaigues, Switzerland). (E) A microscopic view highlights the mesial (M) and distal (D) canal orifices of tooth #37, connected by an isthmus (indicated by a red arrow). (F) A subsequent microscopic image shows the C-shaped root canal of tooth #37, now filled with iRoot SP bioceramic sealer (Innovative Bioceramix, Vancouver, Canada). (G) Master gutta-percha cones are carefully inserted into the mesial and distal canals. (H) A microscopic photograph captures the pulp chamber floor post-completion of the root canal filling. (I) The post-obturation periapical radiograph demonstrates the well-filled C-shaped root canal system in tooth #37, using gutta-percha and bioceramic sealer.

A 23-year-old Chinese female patient visited the Department of Endodontics, reporting chewing pain in her lower left second molar, tooth #37. This pain, rated 3 on the visual analogue scale, had persisted for two weeks. She had not taken any antibiotics or painkillers for relief. The pain was not intense enough to disrupt her sleep. Her medical history was unremarkable, and she was in good general health, classified as ASA I, with no systemic disease symptoms. She maintained good oral hygiene and had no history of harmful or parafunctional habits. Tooth #37 had previously undergone root canal therapy and was fitted with a porcelain-fused-metal crown, but the specifics of this treatment were unknown. Clinical examination showed normal gingiva around the tooth, tenderness upon percussion, and no palpation pain at the root apex. Periodontal probing was within normal limits. A periapical radiograph revealed poor quality in the previous root canal treatment and a radiolucent area around the apex (Figure 1A), suggesting a C-shaped root canal system. The diagnosis was symptomatic apical periodontitis in a previously treated tooth. The porcelain crown's marginal seal was acceptable, but it had partially chipped, exposing the metal base (Figure 1B). The treatment plan included root canal retreatment and a new full crown restoration. The patient declined to replace the existing crown but consented to the retreatment.

Local infiltration anesthesia was administered using 4% articaine with 1:100,000 adrenaline (Septanest; Septodont, Saint-Maru-des-Fosses, France) on both the buccal and lingual sides. The tooth was isolated with a rubber dam (OptiDam; Kerr, Orange, USA). Access was gained through the occlusal surface of the porcelain crown using a dental operating microscope (OPMI PICO, Carl Zeiss, Oberkochen, Germany). Gutta-percha remnants were found in the pulp chamber (Figure 1C) and removed with a #20/.02 Micro-Debrider (Dentsply Maillefer, Ballaigues, Switzerland) (Figure 1D). The mesial and distal canals were identified and negotiated with C-Pilot files (VDW, Munich, Germany). The M3-Pro Nickel Titanium (NiTi) rotary system (United Dental, Changzhou, China) shaped the canals to size #25/.04, with alternating irrigation of 3% sodium hypochlorite (NaOCl) and 17% ethylenediaminetetraacetic acid (EDTA). Ultrasonic tips (Satelec Acteon, Mérignac, France), ET21 and ET25, were used to remove root-filling material in the isthmus connecting the mesial and distal canals. The C-shaped canal configuration was evident (Figure 1E). The irrigation process involved an initial soaking of the canals in a 3% NaOCl solution. This was followed by a series of three irrigation sessions, each lasting 20 seconds, using an Irri-Safe ultrasonic tip (Satelec Acteon). After this, the canals were subjected to a similar irrigation regimen with a 17% EDTA solution, employing the Irri-Safe ultrasonic tip for three distinct 20-second sessions. Once these irrigation steps were completed, the canals were thoroughly cleansed with sterile water and subsequently dried using absorbent paper points. The next step involved the careful introduction of calcium hydroxide paste (Pulpdent™ paste; Pulpdent Corporation, Watertown, USA) into the canals. To conclude the procedure, the access cavity was securely sealed with a temporary filling material, ensuring the integrity of the treatment until the next phase.

One week after the initial treatment, the patient revisited for a follow-up appointment. She reported that the chewing pain had subsided, and tooth #37 was symptom-free. The temporary filling was carefully removed, and the canals were cleansed with 3% NaOCl. The Irri-Safe ultrasonic tip (Satelec Acteon) was then used to eliminate the calcium hydroxide paste. This was followed by a trial fitting of the primary gutta-percha cones. The canals underwent the same irrigation protocol as during the first visit. After irrigation, the canals were dried using paper points. The iRoot SP bioceramic sealer (Innovative Bioceramix, Vancouver, Canada) was then injected into the upper section of the C-shaped root canal system (Figure 1F). The gutta-percha cones were lightly coated with the sealer before insertion into the canal (Figure 1G). These cones were trimmed at the level of the root canal orifice using a heat carrier tip from the Elements Obturation Unit (SybronEndo, Orange, USA) and compacted with a Buchanan plugger (SybronEndo) (Figure 1H). A periapical radiograph was taken to assess the root canal filling, showing a well-obturated C-shaped canal system (Figure 1I). The pulp chamber and the access cavity were then filled using a layering technique with Filtek Z350 XT light-cured composite resin (3M ESPE, St. Paul, USA).

At one-week follow-up, the tooth #37 remained asymptomatic. During follow-up phone calls at three months and one year, the patient, who opted not to attend in-person examinations, reported that tooth #37 remained asymptomatic and functional.

## Discussion

The preoperative assessment of root canal anatomy, especially in retreatment scenarios, is essential for ensuring successful outcomes [29]. In periapical radiographs, mandibular second molars with C-shaped root canal configurations often display a single, conical-shaped root. Fan et al. developed a classification system for the radiographic appearances of mandibular second molars with C-shaped canals, categorizing them into three types based on specific radiographic features [29]. Type I is characterized by a conical or square root with a faint, radiolucent line longitudinally separating the root into distal and mesial sections. This type typically shows a mesial and a distal canal merging into a single canal before exiting at the apical foramen. In our case, the presence of a single conical root in the preoperative radiograph indicated a C-shaped root canal configuration in tooth #37. The radiographic appearance of this tooth's root canal system corresponded to Type I in Fan's classification, suggesting specific considerations for the forthcoming retreatment procedure.

Mandibular second molars with a C-shaped root canal configuration typically exhibit a ribbon-shaped canal interconnected with transverse isthmuses or anastomoses, which often span the entire root length. This complex anatomy poses significant challenges during root canal treatment, particularly at the filling stage. A systematic review and meta-analysis examining the impact of root canal filling quality on periapical lesion status, using CBCT, found that the success of root canal treatment is closely linked to the apical extension of the filling, ideally terminating 0 to 2 mm short of the apical foramen, and to the homogeneity of the filling, which should be gap-free [19]. Kim et al. identified the most frequent causes of treatment failure in mandibular second molars with C-shaped canals as leaky canals and issues with the isthmus [25]. In our specific case, the previously performed root canal filling was suboptimal. The apical extensions of the mesial and distal canals were inadequate, falling 4 mm and 3 mm short of the apical foramen, respectively. Additionally, the homogeneity of the filling was compromised, exhibiting significant gaps along the root canal filling. During the retreatment procedures, the gutta-percha mass was readily visible in the pulp chamber, and a notable absence of sealer was observed in both the pulp chamber and root canals. This observation suggests a severe leakage in the previous root canal filling, likely contributing to the failure of the initial treatment and the development of the periapical lesion in this mandibular second molar. Therefore, the deficiencies in the previous treatment, particularly the poor apical extension and lack of homogeneity in the filling material, are likely the primary reasons for the initial treatment failure and the persistence of the periapical lesion in this case.

Understanding the anatomical features of the pulp chamber floor is crucial in root canal treatment, particularly for accurately locating root canal orifices and identifying complex anatomical structures [30]. This is especially pertinent in mandibular second molars with C-shaped root canals. Min et al. conducted a study using micro-computed tomography to examine the morphology of pulp chamber floors in mandibular second molars with C-shaped canal systems [30]. They reconstructed these floors three-dimensionally and classified them into four types, based on the shape of the pulp chamber floor and the location of dentin fusion between the peninsular-like floor and the pulp chamber wall. Type I is characterized by a peninsula-like floor with a continuous C-shaped orifice. In their study, out of 44 reconstructed pulpal floors, 38 (86.37%) displayed a C-shaped (peninsula-like) floor, among which 8 (18.18%) had a continuous C-shaped orifice (Type I). In our specific case, the pulp chamber floor and the root canal orifice corresponded to Type I (as shown in Figure 1E). This type of pulp chamber floor poses significant challenges in the cleaning, shaping, disinfection, and obturation processes due to the presence of a long isthmus connecting the mesial and distal canals. To address these challenges, a micro-debrider (#20/.02) was employed to extract the gutta-percha points from the root canals and the long isthmus (as depicted in Figure 1C and D). Subsequently, delicate ultrasonic tips, ET21 and ET25, were utilized for the removal of filling material residues in the long isthmus. Moreover, to enhance the effectiveness in removing filling material residues from the C-shaped root canal systems, the Irri-Safe ultrasonic tip was used to activate irrigants (3% NaOCl and 17% EDTA) in three separate 20-second sessions.

The distinctive anatomic characteristic of the C-shaped canal system is the presence of a fin or web that connects the individual root canals. This structure can retain a significant number of microorganisms, even after thorough shaping. Consequently, the application of intracanal medicament is highly recommended in these scenarios to enhance disinfection [31]. During the second visit, the Irri-Safe ultrasonic tip was employed to facilitate the removal of calcium hydroxide paste from the root canal system, simultaneously aiding in further disinfection. 

For the obturation of the C-shaped root canal system, the cold hydraulic technique combined with calcium silicate sealer was chosen. This method is not only convenient but also capable of achieving a root-filling quality comparable to that of warm vertical compaction or lateral compaction with epoxy resin sealer [32]. The effectiveness of this approach was clearly demonstrated in the post-obturation radiograph, showing the successful implementation of the technique and the thorough filling of the complex canal system.

## Conclusions

Retreating a mandibular second molar with a complex C-shaped root canal configuration presents significant challenges. A thorough understanding of the specific root canal anatomy, combined with the use of advanced instruments and techniques, is essential to achieving successful endodontic treatment outcomes.

## References

[1] Haapasalo M, Shen Y, Ricucci D (2008). Reasons for persistent and emerging posttreatment endodontic disease. Endod Topics.

[2] Chen B, Liu H, Wang Z, Ma J, Shen Y (2023). Effects of DJK-5 and chlorhexidine on exopolysaccharide volume and pH in oral biofilms. BMC Oral Health.

[3] Li H, Liu H, Zhang L, Hieawy A, Shen Y (2023). Evaluation of extracellular polymeric substances matrix volume, surface roughness and bacterial adhesion property of oral biofilm. J Dent Sci.

[4] Duncan HF, Kirkevang LL, Peters OA (2023). Treatment of pulpal and apical disease: The European Society of Endodontology (ESE) S3-level clinical practice guideline. Int Endod J.

[5] Li Y, Wang Z, Bao P (2023). Cleaning and disinfecting oval-shaped root canals: ex vivo evaluation of three rotary instrumentation systems with passive ultrasonic irrigation. Medicina (Kaunas).

[6] Lui K, Liu H, Wang H (2023). An application framework of 3D assessment image registration accuracy and untouched surface area in canal instrumentation laboratory research with micro-computed tomography. Clin Oral Investig.

[7] Al-Zuhair H, Su Z, Liu H (2023). Antimicrobial effects of agitational irrigation on single- and multispecies biofilms in dentin canals. Odontology.

[8] Liu H, Nio S, Shen Y (2023). Sodium hypochlorite against Enterococcus faecalis biofilm in dentinal tubules: effect of concentration, temperature, and exposure time. Odontology.

[9] Liu H, Shen Y, Haapasalo M (2023). Effectiveness of six irrigation techniques with sodium hypochlorite in tissue dissolution. Cureus.

[10] Liu H, Shen Y, Wang Z, Haapasalo M (2022). The ability of different irrigation methods to remove mixtures of calcium hydroxide and barium sulphate from isthmuses in 3D printed transparent root canal models. Odontology.

[11] Yang Y, Liu H, Wang ZJ (2023). The effect of acidity on the physicochemical properties of two hydraulic calcium silicate-based cements and two calcium phosphate silicate-based cements. BMC Oral Health.

[12] Yang X, Tian J, Li M (2022). Biocompatibility of a new calcium silicate-based root canal sealer mediated via the modulation of macrophage polarization in a rat model. Materials (Basel).

[13] Zhang W, Liu H, Wang Z, Haapasalo M, Jiang Q, Shen Y (2022). Long-term porosity and retreatability of oval-shaped canals obturated using two different methods with a novel tricalcium silicate sealer. Clin Oral Investig.

[14] Olivieri JG, Encinas M, Nathani T, Miró Q, Duran-Sindreu F (2023). Outcome of root canal retreatment filled with gutta-percha techniques: a systematic review and meta-analysis. J Dent.

[15] Gulabivala K, Ng YL (2023). Factors that affect the outcomes of root canal treatment and retreatment - a reframing of the principles. Int Endod J.

[16] Chugal NM, Clive JM, Spångberg LS (2001). A prognostic model for assessment of the outcome of endodontic treatment: Effect of biologic and diagnostic variables. Oral Surg Oral Med Oral Pathol Oral Radiol Endod.

[17] Chugal NM, Clive JM, Spångberg LS (2003). Endodontic infection: some biologic and treatment factors associated with outcome. Oral Surg Oral Med Oral Pathol Oral Radiol Endod.

[18] Hao J, Liu H, Shen Y (2023). Periapical lesions and missed canals in endodontically treated teeth: a cone-beam computed tomographic study of a Chinese subpopulation. Med Sci Monit.

[19] Alves Dos Santos GN, Faria-E-Silva AL, Ribeiro VL (2022). Is the quality of root canal filling obtained by cone-beam computed tomography associated with periapical lesions? A systematic review and meta-analysis. Clin Oral Investig.

[20] Liu H, Lai WW, Hieawy A, Gao Y, Haapasalo M, Tay FR, Shen Y (2021). Efficacy of XP-endo instruments in removing 54 month-aged root canal filling material from mandibular molars. J Dent.

[21] Liu H, Shabehpour K, Wang Z (2022). Characterisation of deformed or separated nickel-titanium retreatment instruments after clinical use - A multicentre experience: Defect profiles of clinically-used retreatment instruments. J Dent.

[22] Kato A, Ziegler A, Higuchi N, Nakata K, Nakamura H, Ohno N (2014). Aetiology, incidence and morphology of the C-shaped root canal system and its impact on clinical endodontics. Int Endod J.

[23] Fan B, Min Y, Lu G, Yang J, Cheung GS, Gutmann JL (2009). Negotiation of C-shaped canal systems in mandibular second molars. J Endod.

[24] Martins JN, Marques D, Silva EJ, Caramês J, Mata A, Versiani MA (2019). Prevalence of C-shaped canal morphology using cone beam computed tomography - a systematic review with meta-analysis. Int Endod J.

[25] Kim Y, Lee D, Kim DV, Kim SY (2018). Analysis of cause of endodontic failure of C-shaped root canals. Scanning.

[26] Cheung LH, Cheung GS (2008). Evaluation of a rotary instrumentation method for C-shaped canals with micro-computed tomography. J Endod.

[27] Chatchawanwirote Y, Yanpiset K, Jantarat J (2022). Effect of solvent on root canal filling material removal by two different rotary file systems in C-shaped root canals. Aust Endod J.

[28] Gazzaneo I, Amoroso-Silva P, Pacheco-Yanes J (2021). Disinfecting and shaping type I C-shaped root canals: a correlative micro-computed tomographic and molecular microbiology study. J Endod.

[29] Fan B, Cheung GS, Fan M, Gutmann JL, Fan W (2004). C-shaped canal system in mandibular second molars: Part II - radiographic features. J Endod.

[30] Min Y, Fan B, Cheung GS, Gutmann JL, Fan M (2006). C-shaped canal system in mandibular second molars Part III: The morphology of the pulp chamber floor. J Endod.

[31] Chen M, Pradhan B, Meng Y (2024). Micro-computed tomography analysis of calcium hydroxide delivery efficacy in C-shaped canal system of mandibular second molars. BMC Oral Health.

[32] Wisawawatin D, Yanpiset K, Banomyong D, Jantarat J (2023). Gap volume and sealer penetration of C-shaped root canals obturated with cold hydraulic technique and calcium silicate sealer. Aust Endod J.

